# The immunopeptidomic landscape of ependymomas provides actionable antigens for T-cell-based immunotherapy

**DOI:** 10.1093/noajnl/vdae226

**Published:** 2025-01-16

**Authors:** Lena Mühlenbruch, David Rieger, Hannes Becker, Ana Maia Santos Leite, Irina Mäurer, Jens Schittenhelm, Marissa Dubbelaar, Leon Bichmann, Oliver Kohlbacher, Hans-Georg Rammensee, Cécile Gouttefangeas, Marcos Tatagiba, Juliane S Walz, Ghazaleh Tabatabai

**Affiliations:** Cluster of Excellence iFIT (EXC2180) “Image-Guided and Functionally Instructed Tumor Therapies,” Eberhard Karls University Tuebingen, 72076 Tuebingen, Baden-Wuerttemberg, Germany; Department of Peptide-based Immunotherapy, Institute of Immunology, Eberhard Karls University Tuebingen, 72076 Tuebingen, Baden-Wuerttemberg, Germany; Center for Personalized Medicine, Eberhard Karls University Tuebingen, 72076 Tuebingen, Baden-Wuerttemberg, Germany; Center for Neuro-Oncology, Comprehensive Cancer Center Tuebingen-Stuttgart, University Hospital Tuebingen, Eberhard Karls University Tuebingen, 72076 Tuebingen, Baden-Wuerttemberg, Germany; Department of Neurology and Interdisciplinary Neuro-Oncology, University Hospital Tuebingen, Hertie Institute for Clinical Brain Research, Eberhard Karls University Tuebingen, 72076 Tuebingen, Baden-Wuerttemberg, Germany; Cluster of Excellence iFIT (EXC2180) “Image-Guided and Functionally Instructed Tumor Therapies,” Eberhard Karls University Tuebingen, 72076 Tuebingen, Baden-Wuerttemberg, Germany; Department of Neurosurgery, University Hospital Tuebingen, Eberhard Karls University Tuebingen, 72076 Tuebingen, Baden-Wuerttemberg, Germany; Center for Personalized Medicine, Eberhard Karls University Tuebingen, 72076 Tuebingen, Baden-Wuerttemberg, Germany; Center for Neuro-Oncology, Comprehensive Cancer Center Tuebingen-Stuttgart, University Hospital Tuebingen, Eberhard Karls University Tuebingen, 72076 Tuebingen, Baden-Wuerttemberg, Germany; Department of Neurology and Interdisciplinary Neuro-Oncology, University Hospital Tuebingen, Hertie Institute for Clinical Brain Research, Eberhard Karls University Tuebingen, 72076 Tuebingen, Baden-Wuerttemberg, Germany; Institute for Immunology, Eberhard Karls University Tuebingen, 72076 Tuebingen, Baden-Wuerttemberg, Germany; Cluster of Excellence iFIT (EXC2180) “Image-Guided and Functionally Instructed Tumor Therapies,” Eberhard Karls University Tuebingen, 72076 Tuebingen, Baden-Wuerttemberg, Germany; Center for Personalized Medicine, Eberhard Karls University Tuebingen, 72076 Tuebingen, Baden-Wuerttemberg, Germany; Center for Neuro-Oncology, Comprehensive Cancer Center Tuebingen-Stuttgart, University Hospital Tuebingen, Eberhard Karls University Tuebingen, 72076 Tuebingen, Baden-Wuerttemberg, Germany; Department of Neurology and Interdisciplinary Neuro-Oncology, University Hospital Tuebingen, Hertie Institute for Clinical Brain Research, Eberhard Karls University Tuebingen, 72076 Tuebingen, Baden-Wuerttemberg, Germany; German Cancer Consortium (DKTK), Partner Site Tuebingen, 72076 Tuebingen, Baden-Wuerttemberg, Germany; Department of Neuropathology, University Hospital Tuebingen, Eberhard Karls University Tuebingen, 72076 Tuebingen, Baden-Wuerttemberg, Germany; Center for Personalized Medicine, Eberhard Karls University Tuebingen, 72076 Tuebingen, Baden-Wuerttemberg, Germany; Center for Neuro-Oncology, Comprehensive Cancer Center Tuebingen-Stuttgart, University Hospital Tuebingen, Eberhard Karls University Tuebingen, 72076 Tuebingen, Baden-Wuerttemberg, Germany; Quantitative Biology Center (QBiC), Eberhard Karls University Tuebingen, 72076 Tuebingen, Baden-Wuerttemberg, Germany; Institute for Immunology, Eberhard Karls University Tuebingen, 72076 Tuebingen, Baden-Wuerttemberg, Germany; Cluster of Excellence iFIT (EXC2180) “Image-Guided and Functionally Instructed Tumor Therapies,” Eberhard Karls University Tuebingen, 72076 Tuebingen, Baden-Wuerttemberg, Germany; Department of Peptide-based Immunotherapy, Institute of Immunology, Eberhard Karls University Tuebingen, 72076 Tuebingen, Baden-Wuerttemberg, Germany; Applied Bioinformatics, Department of Computer Science, Eberhard Karls University Tuebingen, 72076 Tuebingen, Baden-Wuerttemberg, Germany; Institute for Immunology, Eberhard Karls University Tuebingen, 72076 Tuebingen, Baden-Wuerttemberg, Germany; Institute for Bioinformatics and Medical Informatics, University of Tuebingen, 72076 Tuebingen, Baden-Wuerttemberg, Germany; Institute for Translational Bioinformatics, University Hospital Tuebingen, 72076 Tuebingen, Baden-Wuerttemberg, Germany; Cluster of Excellence Machine Learning in the Sciences (EXC2064), University of Tuebingen, 72076 Tuebingen, Baden-Wuerttemberg, Germany; Applied Bioinformatics, Department of Computer Science, Eberhard Karls University Tuebingen, 72076 Tuebingen, Baden-Wuerttemberg, Germany; German Cancer Consortium (DKTK), Partner Site Tuebingen, 72076 Tuebingen, Baden-Wuerttemberg, Germany; Institute for Immunology, Eberhard Karls University Tuebingen, 72076 Tuebingen, Baden-Wuerttemberg, Germany; Cluster of Excellence iFIT (EXC2180) “Image-Guided and Functionally Instructed Tumor Therapies,” Eberhard Karls University Tuebingen, 72076 Tuebingen, Baden-Wuerttemberg, Germany; German Cancer Consortium (DKTK), Partner Site Tuebingen, 72076 Tuebingen, Baden-Wuerttemberg, Germany; Institute for Immunology, Eberhard Karls University Tuebingen, 72076 Tuebingen, Baden-Wuerttemberg, Germany; Cluster of Excellence iFIT (EXC2180) “Image-Guided and Functionally Instructed Tumor Therapies,” Eberhard Karls University Tuebingen, 72076 Tuebingen, Baden-Wuerttemberg, Germany; German Cancer Consortium (DKTK), Partner Site Tuebingen, 72076 Tuebingen, Baden-Wuerttemberg, Germany; Department of Neurosurgery, University Hospital Tuebingen, Eberhard Karls University Tuebingen, 72076 Tuebingen, Baden-Wuerttemberg, Germany; Center for Personalized Medicine, Eberhard Karls University Tuebingen, 72076 Tuebingen, Baden-Wuerttemberg, Germany; Center for Neuro-Oncology, Comprehensive Cancer Center Tuebingen-Stuttgart, University Hospital Tuebingen, Eberhard Karls University Tuebingen, 72076 Tuebingen, Baden-Wuerttemberg, Germany; Clinical Collaboration Unit Translational Immunology, German Cancer Consortium (DKTK), Department of Internal Medicine, University Hospital Tuebingen, 72076 Tuebingen, Baden-Wuerttemberg, Germany; German Cancer Consortium (DKTK), Partner Site Tuebingen, 72076 Tuebingen, Baden-Wuerttemberg, Germany; Cluster of Excellence iFIT (EXC2180) “Image-Guided and Functionally Instructed Tumor Therapies,” Eberhard Karls University Tuebingen, 72076 Tuebingen, Baden-Wuerttemberg, Germany; Department of Peptide-based Immunotherapy, Institute of Immunology, Eberhard Karls University Tuebingen, 72076 Tuebingen, Baden-Wuerttemberg, Germany; German Cancer Consortium (DKTK), Partner Site Tuebingen, 72076 Tuebingen, Baden-Wuerttemberg, Germany; Center for Personalized Medicine, Eberhard Karls University Tuebingen, 72076 Tuebingen, Baden-Wuerttemberg, Germany; Center for Neuro-Oncology, Comprehensive Cancer Center Tuebingen-Stuttgart, University Hospital Tuebingen, Eberhard Karls University Tuebingen, 72076 Tuebingen, Baden-Wuerttemberg, Germany; Department of Neurology and Interdisciplinary Neuro-Oncology, University Hospital Tuebingen, Hertie Institute for Clinical Brain Research, Eberhard Karls University Tuebingen, 72076 Tuebingen, Baden-Wuerttemberg, Germany; Cluster of Excellence iFIT (EXC2180) “Image-Guided and Functionally Instructed Tumor Therapies,” Eberhard Karls University Tuebingen, 72076 Tuebingen, Baden-Wuerttemberg, Germany

**Keywords:** cancer vaccination, HLA ligands, immunotherapy, mass spectrometry, peptide vaccine

## Abstract

**Background:**

Ependymoma are primary tumors of the nervous system. Due to their growth pattern, many ependymomas can be managed with neurosurgical resection alone. A substantial proportion of these tumors recurs or displays infiltrative growth patterns. Further established therapeutic options include radiation therapy. Systemic treatment options include platinum-based therapeutic regimes or a combination of lapatinib and temozolomide. Peptide-based immunotherapy represents a promising therapeutic strategy relying on the induction of tumor-specific T cells targeting human leukocyte antigens (HLA)-presented peptides. Our work aimed to analyze the landscape of naturally presented HLA class I and II ligands of primary ependymomas (EPN) to delineate EPN-associated antigens.

**Methods:**

We investigated 22 EPN tissue samples using a comparative mass spectrometry-based immunopeptidomic approach. Additionally, EPN-specific antigens were functionally characterized in T-cell-based immunogenicity assays.

**Results:**

We discovered a subset of EPN-exclusive peptides including HLA-A*02 and HLA-A*25/HLA-A*26–restricted HLA ligands and identified a small panel of cancer/testis antigens (CTAs)-derived HLA ligands. Furthermore, we outlined immunopeptidomic alterations in different ependymoma subgroups and progressive ependymoma. Subsequently, we performed functional characterization of the previously identified HLA-A*02:01 restricted peptide FLDS to demonstrate immunogenicity in vitro.

**Conclusion:**

The immunopeptidome landscape of EPNs provides actionable targets that could further be explored as a T cell-based immunotherapeutic strategy in this tumor entity.

**Key Points**

- The immunopeptidome landscape of EPNs contains entity-exclusive antigens.
- EPN-specific peptides can lead to detectable, spontaneous EPN-specific T cell responses.
- EPN-specific peptides might contribute to the design of T cell-based immunotherapeutic strategies.

**Importance of the study**

This study demonstrates that the immunopeptidome landscape of ependymomas provides actionable targets. These data can pave the way for the design and further investigations of T cell-based immunotherapeutic strategy in this tumor entity. Established postsurgical treatment strategies for ependymomas currently include radiation therapy, platinum-based therapeutic regimes, or a combination of lapatinib and temozolomide. Peptide-based cancer vaccines rely on the induction of tumor-specific T cells targeting human leukocyte antigens (HLA)-presented peptides and thus offer a new therapeutic strategy for ependymoma. We present a subset of ependymoma-exclusive peptides including HLA-A*02 and HLA-A*25/HLA-A*26–restricted HLA ligands. We performed a functional characterization of the previously identified HLA-A*02:01 restricted peptide FLDS to demonstrate immunogenicity in vitro.

Ependymomas (EPNs) are primary neuroepithelial tumors of the central nervous system (CNS) that arise from ependymal cells. They may occur in the context of neurofibromatosis 2 (NF2)-associated schwannomatosis or as sporadic tumors along the entire neuroaxis.^1^ EPNs are rare, accounting for up to 3% of all primary tumors of the CNS.^2^ According to the latest publication of the Central Brain Tumor Registry of the USA, the annual incidence rate is 0.52 per 100 000 persons.^3^

In adult patients, EPNs are predominantly located in the spinal cord, whereas pediatric EPNs mostly occur intracranially, either in a supratentorial location or in the posterior cranial fossa.^4–6^ Comprehensive molecular profiling has profoundly shaped the biological subgrouping of EPNs.^7–9^ In fact, the current WHO classification has substantially revised the diagnostic criteria for EPNs,^1^ based on these studies and according to the seventh recommendation of the cIMPACT-NOW consortium.^10^ It subdivides EPNs into various subtypes using DNA methylation profiling.^9^ The integrated molecular diagnosis and grading in three grades of malignancy (CNS WHO grades 1-3) is based on neuroanatomic location (supratentorial, posterior fossa, spinal cord), as well as molecular characteristics specific for each compartment.^11^ The prognosis of EPN patients is affected by many factors, such as localization, age at diagnosis, molecular profile and the extent of resection. Pediatric EPNs have a poorer prognosis and higher recurrence rates than adult EPNs.^5,12^

Neurosurgical intervention is the most relevant therapeutic strategy for EPN patients, followed by radiation therapy for selected cases, for example upon subtotal resection, progression after surgery, or for EPNs with infiltrative growth patterns. Systemic treatment options are not established and are usually defined by the interdisciplinary tumor board for each individual case.^13,14^ Recent data from precision oncology approaches indicate that some EPNs harbor actionable somatic molecular alterations, for example activation of the mTOR signaling pathway.^15,16^ Furthermore, genetic alterations such as gene fusions involving zinc finger translocation-associated (ZFTA) or yes-associated protein 1 (YAP1) and histone H3, enhancer of zeste homolog inhibitory protein (EZHIP), or telomerase reverse transcriptase (TERT) mutations may be considered as druggable targets.^1^ There remains an unmet medical need for effective treatment options for EPN patients that cannot be controlled by neurosurgery and radiotherapy alone.

Immunotherapy represents a promising strategy for EPN patients. Previous studies showed a significant expression of the immune-checkpoint protein Programmed cell death 1 ligand 1 (PD-L1) as well as a substantial infiltration of Cluster of Differentiation (CD) 8^+^ cytotoxic T cells in EPNs.^17,18^ Therefore, immunotherapeutic strategies with immune checkpoint inhibitors (ICI) could be promising therapeutic approaches in EPNs.^19^ Some case reports revealed therapeutic responses to immune checkpoint inhibition in EPNs with prolonged stable disease.^20–22^ Furthermore, several cell surface markers (EPHA2, Il-13Ra2, and HER2) were identified as promising targets for immunotherapeutic strategies with CAR T cells.^23^ However, a landscape of actionable antigens in EPNs has not yet been defined.

In this study, we characterized the human leukocyte antigen (HLA) immunopeptidome of adult EPNs using liquid chromatography-mass spectrometry (LC-MS/MS) for the discovery of potential immunotherapeutic targets. Comparative profiling with immunopeptidomes of benign tissue reference database and glioblastoma (GBM) revealed a panel of tumor-associated peptides, cancer/testis antigen (CTA)-derived HLA ligands, as well as subgroup-associated differences and alterations in case of tumor progression. Additional immunogenicity testing indicated spontaneous, ependymoma-specific T cell responses in EPN patients. Based on these investigations, we provide actionable targets for T-cell-based immunotherapy for this tumor entity.

## Methods

### Patients

The study cohort consisted of 22 EPN tissue samples derived from 21 different donor patients (10 intracranial EPNs, 12 EPNs of the spinal cord). The median age at the time of resection was 42.5 years. EPN tumors were surgically resected, immediately snap-frozen in liquid nitrogen (N_2_), and subsequently stored at −80 °C. Informed written consent was obtained in accordance with the Declaration of Helsinki protocol. The study was performed according to the guidelines of the local ethics committee (332/2017BO2). Patient characteristics and diagnoses are provided in Supplementary Table S1.

### Isolation of HLA-presented peptides

HLA class I- and class II-presented peptides were isolated from tissue samples performing standard immunoaffinity purification as previously described in ^[12]^. The pan-HLA class I-specific mouse monoclonal antibody (mAb) W6/32, the pan-HLA class II-specific mAb Tü-39, and the HLA-DR-specific mAb L243 (all produced in-house) were used to extract HLA molecules.

### Analysis of HLA ligands by liquid chromatography-tandem MS

HLA ligand extracts were analyzed as described before.^24^ HLA ligands were separated using reversed-phase ultra-high performance liquid chromatography (nanoUHPLC, UltiMate 3000 RSLCnano, Dionex). Eluted peptides were analyzed by tandem MS in an on-line coupled LTQ Orbitrap XL hybrid mass spectrometer (Thermo Fisher Scientific) equipped with a nano-electron spray ion source.

### Data processing

Data processing was performed using the Proteome Discoverer 1.4 software (Thermo Fisher Scientific). Database search and spectral annotation were performed against the human proteome as comprised in the UniProtKB/Swiss-Prot database (20 279 reviewed protein sequences; September 27th, 2013; www.uniprot.org) *via* the SequestHT algorithm. Mass tolerance for processing was set to 5 ppm for precursor ions and 0.5 Da for fragment ions. Oxidized methionine was allowed as only dynamic modification, and no cleavage specificity was selected. Peptide confidence was determined using the Percolator algorithm 2.04^25^ with a target value of *q* ≤ 0.05 (5% FDR). After processing, additional filters for search engine rank (=1) and peptide length (=8-25 amino acids) were applied. HLA class I ligand annotation was performed using SYFPEITHI^26^ and NetMHCpan 4.0.^27^

### Software, statistical analysis, and online tools

Statistical analysis was performed using GraphPad Prism versions 6.1, 8.2.1, and 9.0.1 software (GraphPad Software Inc). For overlapping analysis, the bioinformatics tools BioVenn^28^ and jVenn^29^ were used. The benign reference dataset used for comparative profiling was comprised of the immunopeptidome data of a previously reported hematological benign cohort,^30^ the benign tissue dataset provided within the HLA Ligand Atlas,^31^ as well as additional in-house acquired immunopeptidome data of benign tissue and cell line samples. This benign reference dataset included the immunopeptidomic data of 12 brain samples. The database Genotype-Tissue Expression Project (GTEx; www.gtexportal.org)^25^ was consulted for mRNA expression profiles of source proteins in healthy tissue to exclude brain-specific antigens (median mRNA expression level in benign tissue except testis ≤ 15 TPM). Gene ontology (GO) term enrichment analyses were performed with the Panther 16.0 database (Released February 1, 2020) with the integrated “statistical overrepresentation test” (Release July 11, 2019).^32^ Gene identifiers of ligandomic source proteins shared by EPNs and GBMs were queried against the “GO biological process complete” database using the default “*Homo sapiens* genes” reference list. GO terms were sorted by Fisher’s exact raw *P*-value, and the top 5 scoring terms were reported as overrepresented. Their corresponding *P*-values were selected for illustration.

### Peptide synthesis

Synthetic peptides were produced using the Liberty Blue Automated Peptide Synthesizer (CEM) by applying the 9-fluorenylmethyl-oxycarbonyl/tert-butyl strategy as previously described.^34^ For the immunogenicity assays, lyophilized peptides FLDSQITTV (HLA-A*02:01 binder found on EPN-018, source protein CF299, abbrev.: FLDS) as well as EVLNGQVSKY (source protein DYH6, abbrev.: EVLN) and ETVDENGRLY (source protein CCD13, abbrev.: ETVD), both HLA-A*25:01/HLA-A*26:01 binders found on EPN-014 (Supplementary Table S3), were solved in milliQ water containing 10% DMSO at 1 mg/mL, aliquoted, and frozen at −80 °C until use.

### Immunogenicity testing

Peripheral blood mononuclear cells (PBMCs) were isolated from Lithium Heparin blood by cell density gradient centrifugation (Biocoll, Merck, Darmstadt, Germany), washed twice in PBS, counted using the NucleoCounter NC-250 (Chemometec, Allerod, Denmark) and frozen at 9-12.5 × 10^6^ cells/cryovial in 90% heat-inactivated (h.i.) fetal bovine serum (FBS, Thermofisher Scientific, Waltham, MA, USA) and 10% DMSO (Merck). Cryovials were placed at −80 °C in a freezing container (Mr. Frosty, Merck) and transferred to liquid nitrogen for long-term storage.

Blood from healthy donors was collected from lithium heparin tubes obtained or from cones (healthy donors, HDs) from the Transfusion Medicine Department (Tübingen, projects 156/2012BO1 and 713/2018BO2).

For in vitro short-term cultures, PBMCs were thawed in IMDM (Lonza, Basel, Switzerland) containing 2.5% h.i. human serum (Capricorn, Ebsdorfergrund, Germany), 100-U/mL penicillin, 100-μg/mL streptomycin (Sigma-Aldrich, St. Louis, MO, USA), and 50-μM β-mercaptoethanol (Merck) containing 3-μg/mL DNAse I (Sigma-Aldrich), counted, and seeded in T cell medium (TCM = IMDM with 10% h.i. human serum, 100-U/mL penicillin, 100-μg/mL streptomycin, and 50 μM β-mercaptoethanol). Peptide stimulation was performed at 37 °C and 7.5 % CO_2_ over a 12-day period as described previously.^35,36^ Briefly, 5-μg/mL peptide were added on day 1 and rhIL2 (R&D Systems, Minneapolis, NM) was added on days 3, 5, 7, and 9. Cells were split if needed and at day 12, harvested, washed, counted, and tested.

IFN-γ ELISpot was performed in 4 replicates per condition using 5-μg/mL synthetic peptides. Final concentration of DMSO in the well was matched between the CTRL^-^ and the peptide wells (both 200.000 cells/well), or phytohemagglutinin-L (PHA-L) as positive control (CTRL^+^, 100.000 cells/well). Cells in TCM were stimulated for 26 h at 37 °C and 7.5 % CO_2_; then, the ELISpot plate was revealed according to the published protocol.^36,37^ Spots were counted with the ImmunoSpot series 6 ultra-V analyzer (CTL, Cleveland, OH).

For MHC tetramer staining, an HLA-A*0201 monomer containing the peptide FLDS was produced by UV-exchange of a conditional ligand,^38^ tetramerized with Streptavidin PE (Thermofisher, Waltham, MA), aliquoted, and frozen at −80 °C in the presence of glycerol.^39^ For staining, the tetramer was diluted to 5 μg/mL in PBS containing 50% h.i. FBS, 2 mM EDTA, 0.02% NaN_3_ and added on 1 × 10^6^ cells for 30 min at room temperature in the dark. After one wash in FACS buffer (PBS with 2% h.i. FBS, 2 mM EDTA and 0.02% NaN_3_), cells were stained with the following antibodies (Ab): CD4 FITC (clone HP2/6, in-house production) and CD8 PE-Cy7 (clone SFCI21Thy2D3, Beckman Coulter, Brea, CA), and with the dead/live marker Zombie Aqua (Biolegend, San Diego, CA) at pretested dilutions for an additional 20 min at 4 °C in the dark before final washing.

For intracellular cytokine staining, cells were cultured for 14 h at 37 °C and 7.5 % CO_2_ with 10-µg/mL peptide or equivalent 10% DMSO as negative control (CTRL^-^), in the presence of 10-µg/mL Brefeldin A (Sigma-Aldrich) and GolgiStop (Beckton Dickinson, Heidelberg, Germany). Staphylococcus enterotoxin B (Sigma-Aldrich) was used as positive control (data not shown). Staining was performed in three steps as described,^35,36^ using Abs CD107a FITC (clone H4A3, Becton Dickinson, Heidelberg, Germany; 1.5 µL/test added during the culture), CD8 PE-Cy7, CD4 APC-Cy7 (clone RPA-T4), IFN-γ BV421 (clone B27), TNF BV605 (clone Mab11), and CD154 APC (clone 24-31) and Zombie Aqua at pretested dilutions (all Biolegend). A T cell response was defined if CD107a or cytokine expression increased at least 3-fold in the peptide stimulated condition as compared with the CTRL^-^.

Cells were acquired on an LSR FortessaTM SORP flow cytometer (Becton Dickinson, New Jersey, USA) equipped with DIVA (Version 6.1.3) and data analysis was performed with the FlowJo software (Version 10.6.1).

## Results

### MS-based identification of naturally presented EPN-associated HLA class I ligands

**Figure 1.**
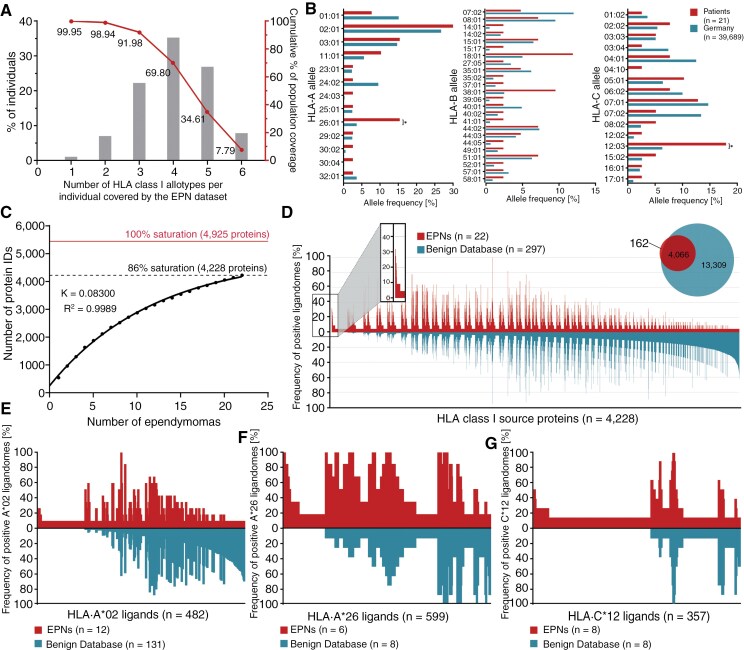
HLA class I ligandome analysis and identification of EPN-associated HLA ligands (A) HLA class I allotype population coverage within the EPN patient cohort compared with the world population (calculated by the IEDB population coverage tool, www.iedb.org).^40^ (B) HLA class I allelic distribution in the EPN patient cohort and a German reference population (“Germany pop 8,” Allele Frequency Net Database AFND, www.allelefrequencies.net).^41^ (C) Saturation analysis of HLA class I ligand source proteins of the EPN patient cohort. Number of unique source protein identifications is shown as a function of cumulative immunopeptidome analysis of EPN samples (*n* = 22). Exponential regression allowed for the robust calculation (*R*^2^ = 0.9989) of the maximum attainable number of different source protein identifications (red line). The dashed line depicts the source proteome coverage achieved in the EPN patient cohort. (D) Comparative profiling of HLA class I source proteins based on the frequency of HLA-restricted presentation in EPNs and benign ligandomes. Frequencies of positive immunopeptidomes for the respective source proteins (*x*-axis) are indicated on the *y*-axis. Comparative profiling of (E) HLA-A*02 ligands, (F) HLA-A*26 ligands, and (G) HLA-C*12 ligands. *P-value ≤ .05 after Bonferroni correction; EPN, ependymoma.

The EPN patient study cohort (*n* = 21) comprised a total of 53 different HLA class I allotypes, covering at least 1 HLA class I allotype for 99.95% of individuals within the world’s population (Figure 1A).^40,42^ Most frequent alleles within the patient cohort were HLA-A*02:01 (*n* = 12), HLA-A*03:01 (*n* = 6), HLA-A*26:01 (*n* = 6), HLA-B*18:01 (*n* = 5), HLA-C*07:01 (*n* = 5), and HLA-C*12:03 (*n* = 7). The HLA class I allelic distribution was representative for most alleles in comparison to a German reference population (cohort “Germany pop 8” (*n* = 39 689); www.allelefrequencies.net).^41^ In contrast, allele frequencies of HLA-A*26:01 (15.4% and 3.6%; *P*-value < .0001; OR = 4.9; 95% CI = 2.0 to 11.6) and HLA-C*12:03 (17.9% and 6.3%; *P-*value = .0028; OR = 3.3; 95% CI = 1.4 to 7.4) were significantly increased in the EPN patient cohort compared with the reference cohort (Figure 1B).

LC-MS/MS-based analysis of the HLA class I ligandomes isolated from 22 EPN tissue samples from 21 patients identified a total of 6185 unique HLA class I ligands (range: 27-1340 peptides per sample; mean: 485 peptides per sample) derived from 4228 different source proteins (Supplementary Figure S1A and Supplementary Table S2), obtaining 86% of the estimated maximum attainable coverage in HLA ligand source proteins (Figure 1C). A positive correlation between tissue sample masses and yields of HLA class I ligands was observed (*P*-value = .0191; Pearson´s correlation coefficient *r* = .4951; 95% CI = 0.1 to 0.8) (Supplementary Figure S1B). As expected, the majority of HLA class I ligands (71%) exhibited a peptide length of 9 amino acids (Supplementary Figure S1D).

To identify EPN-associated antigens, a comparative HLA class I ligandome profiling of the EPN cohort was performed against a benign reference database. This database mainly comprised the immunopeptidome data of a previously reported hematological benign cohort^30^ and the benign tissue dataset provided within the HLA Ligand Atlas.^31^ Altogether, the benign reference database contained HLA class I ligandome data from 33 hematological and nonhematological entities (*n* = 419) comprising a total of 152 729 unique HLA class I-presented peptides derived from 17 173 different source proteins. Overlap analysis between the EPNs and the benign reference datasets revealed 162 HLA class I ligand source proteins that were presented exclusively on EPN samples (Figure 1D) and were never detected on benign samples. Three of these tumor-exclusive source proteins were identified with a representation frequency ≥ 10% (≥3 samples) among the EPN patients.

To identify EPN-associated HLA ligands with high representation frequencies, HLA allotype-specific immunopeptidome profiling for the most common HLA allotype restrictions of the EPN-exclusive HLA class I ligands (HLA-A*02, HLA-A*26, and HLA-C*12) was performed (Figure 1E–G). This resulted in the identification of 5 HLA-A*02-, 41 HLA-A*26-, and 24 HLA-C*12-restricted ligands with representation frequencies ≥17%, 33%, and 25% (≥2 samples), respectively. Further validation of EPN-exclusive HLA ligands included on peptide level systematic auditing of MS spectrum quality as well as on source protein level exclusion of corresponding mRNA expression in healthy (brain) tissue through consultation of the database GTEx^43^ (Supplementary Data S1). Thereby, at a target-definition FDR < 5% (Supplementary Figure S1F), a panel of 6 EPN-exclusive peptides was selected as potentially EPN-associated HLA ligands, including 1 HLA-A*02–restricted and 5 HLA-A*25/HLA-A*26–restricted peptides (Supplementary Table S3).

### HLA class II ligandome profiling

**Figure 2.**
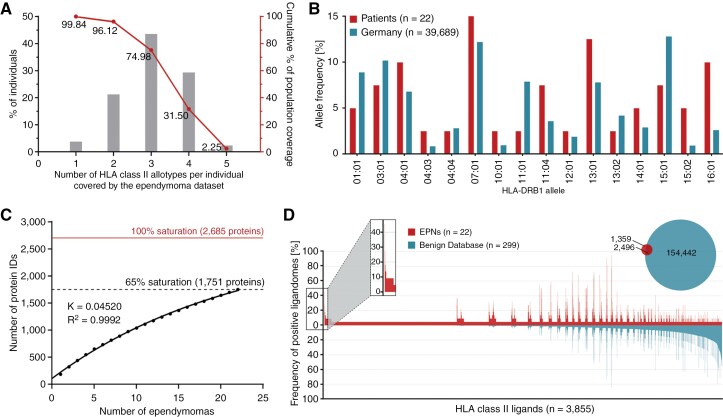
**HLA class II ligandome profiling.** (A) HLA class II allotype population coverage within the EPN patient cohort compared with the world population (calculated by the IEDB population coverage tool, www.iedb.org).^40^ (B) HLA class II allelic distribution in the EPN patient cohort and a German reference population (“Germany pop 8,” Allele Frequency Net Database AFND, www.allelefrequencies.net).^41^ (C) Saturation analysis of source proteins of HLA class II-presented peptides from the EPN patient cohort. Number of unique source protein identifications is shown as a function of cumulative immunopeptidome analysis of EPN samples (*n* = 22). Exponential regression allowed for the robust calculation (*R*^2^ = 0.9992) of the maximum attainable number of different source protein identifications (red line). The dashed line depicts the source proteome coverage achieved in the EPN patient cohort. (D) Comparative profiling of HLA class II-presented peptides based on the frequency of presentation in EPNs and benign ligandomes. Frequencies of positive immunopeptidomes for the respective source proteins (*x*-axis) are indicated on the *y*-axis. *P-value ≤ .05 after Bonferroni correction; EPN, ependymoma.

Sixteen different HLA-DRB1 and 13 different HLA-DQB1 allotypes were comprised in the EPN patient cohort, covering at least 1 HLA class II allotype in 99.84% of individuals among the world’s population (Figure 2A). The most frequent alleles within the EPN patient cohort were HLA-DRB1*07:01 (*n* = 6), HLA-DRB1*13:01 (*n* = 5), HLA-DQB1*03:01 (*n* = 5), and HLA-DQB1*06:03 (*n* = 5). Comparison of allele frequencies within the EPN patient cohort with those of a German reference population (cohort “Germany pop 8” (*n* = 39 689, www.allelefrequencies.net)^41^ revealed no significant differences in HLA class II allelic distribution (Figure 2B).

Mapping the HLA class II ligandomes of 22 EPN tissue samples by LC-MS/MS revealed a total of 3855 unique HLA class II-presented peptides (range: 83-1127 peptides per sample; mean: 263 peptides per sample) from 1751 source proteins (Supplementary Figure S1A; Supplementary Table S2), achieving a 65% coverage of the estimated maximum attainable number of source proteins (Figure 2C). A positive correlation of sample masses and yields of HLA class II-presented peptides was shown (*P*-value = .0002; Pearson´s correlation coefficient *r* = 0.7112; 95% CI = 0.4 to 0.9) (Supplementary Figure S1C). Lengths of HLA class II-presented peptides were distributed across the tolerated range of 9-25 amino acids, with 15 amino acids as the most abundant peptide length (18%) (Supplementary Figure S1E).

Comparative HLA class II ligandome profiling of the EPN cohort against a benign reference dataset^30,31^ comprising immunopeptidomic data from 32 hematological and nonhematological entities (*n*(samples) = 364) with a total of 218 251 unique HLA class II-presented peptides derived from 15 959 source proteins, was performed to identify EPN-exclusive HLA class II-presented peptides. Overlap analysis revealed 1359 EPN-exclusive HLA class II-presented peptides (Fig. 2D), and 11 of these tumor-exclusive HLA class II-presented peptides were identified with a representation frequency ≥ 10% (≥3 samples) among the patients. However, at a target-definition FDR < 5% (Supplementary Figure S1G), no peptide withstood detailed validation on peptide and source protein level evaluating MS spectra quality and mRNA expression profiles in healthy tissue.^43^

Thus, HLA class II ligandome profiling provided no further potentially tumor-associated HLA-presented peptides in addition to the already selected HLA class I ligands.

### EPNs share HLA-presented antigens with GBMs

**Figure 3.**
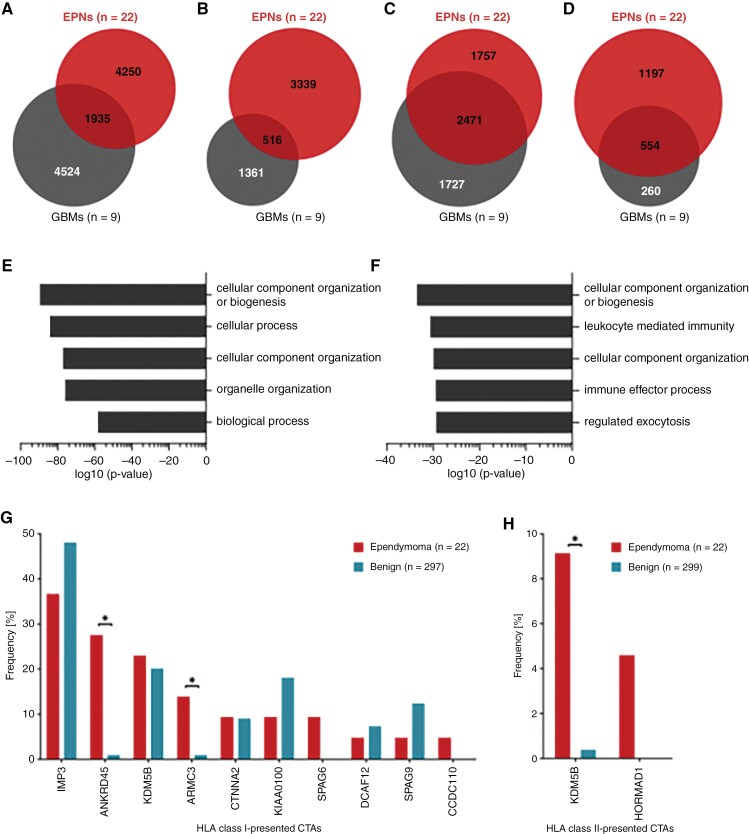
**Comparison of EPN and GBM HLA class I ligandomes and identification of CTAs in the EPN** **immunopeptidome.** Overlap analysis was performed with (A) HLA class I ligands, (B) HLA class II-presented peptides, (C) HLA class I ligand source proteins, and (D) source proteins of HLA class II-presented peptides identified in the EPN cohort and those identified in a previously published GBM cohort (*n* = 9).^44^ GO term enrichment of biological processes was performed using the panther DB webservice^32^ with (E) HLA class I ligand source proteins and (F) source proteins of HLA class II-presented peptides shared by EPNs and GBMs. Top 5 enriched terms with respect to their log10 *P*-value (Fisher’s exact test) were selected. Representation frequencies of published CTAs^45^ in (G) HLA class I and (H) HLA class II ligandomes of ependymomas and in the benign reference immunopeptidomes. Fisher’s exact test was applied for comparative statistical analysis. *P-value ≤ .05 after Bonferroni correction; CTA, cancer/testis antigen; EPN, ependymoma; GBM, glioblastoma.

Comparative analysis between the immunopeptidome of the EPNs (*n* = 22) and a previously published GBM (*n* = 9) dataset^44^ revealed 31% of EPN HLA class I ligands and 13% of EPN HLA class II-presented peptides shared by both entities (Figure 3A and B). The overlap was larger on the source protein level, with 58% of EPN HLA class I source proteins also identified in GBMs (Figure 3C). However, all source proteins of the 6 EPN-associated HLA class I ligands showed EPN-exclusive representation. Furthermore, 32% of the EPN HLA class II-presented antigens were also identified in the immunopeptidome of GBMs (Figure 3D). GO term enrichment of biological processes (panther DB web service^32^) was performed to investigate the functions of shared antigenic source proteins. For HLA class I antigens, an enrichment in proteins involved in cellular component and organelle organization was revealed (Figure 3E). For shared HLA class II antigens, cellular component organization was observed, as well as an involvement in immunological processes such as leukocyte-mediated immunity and immune effector processes (Figure 3F).

Altogether, EPNs and GBMs share a large set of HLA-presented antigens. However, the two tumor entities also display differences on immunopeptidomic level through entity-exclusive antigens that might be crucial for the selection of potential immunotherapeutic targets.

### Cancer/testis antigens-derived HLA ligands are identified in the immunopeptidome of EPNs

In addition to the identification of novel EPN-associated HLA ligands, immunopeptidomes of EPNs were analyzed for the presence of CTA^45^-derived HLA ligands. We identified 19 different HLA class I ligands and 2 HLA class II-presented peptides that derived from 10 and 2 CTAs, respectively (Supplementary Table S4). HLA class I ligands from 4 CTAs were identified in more than 10% of the EPN samples, comprising U3 small nucleolar ribonucleoprotein IMP3 (IMP3) represented by 3 different peptides in 8 tumor samples, the ankyrin repeat domain-containing protein 45 (ANKRD45), of which the same peptide was found in 6 different samples, and the armadillo repeat-containing protein 3 (ARMC3) presented by 2 different peptides in 3 samples. Another frequently identified CTA was the lysine-specific demethylase 5B (KDM5B), which was found as source protein of 4 HLA class I ligands and 1 HLA class II-presented peptide. However, most identified CTAs were lacking tumor-exclusive representation with the respective HLA ligands identified in EPNs as well as in benign immunopeptidomes (Figure 3G and H). The analysis delineated only a small panel of 4 EPN-exclusive, but infrequent, HLA ligands derived from 3 CTAs (SPAG6, CCDC110, and HORMAD1). Still, the HLA class I-presented CTAs ANKRD45 and ARMC3 as well as the HLA class II-presented CTA KDM5B were identified more frequently in the EPN cohort than in the benign reference dataset (Figure 3E and F).

Hence, EPN immunopeptidomes comprise HLA class I ligands and HLA class II-presented peptides derived from previously described CTAs, some of which bear potential as immunotherapeutic targets.

### Tumor subgroup analysis

**Figure 4.**
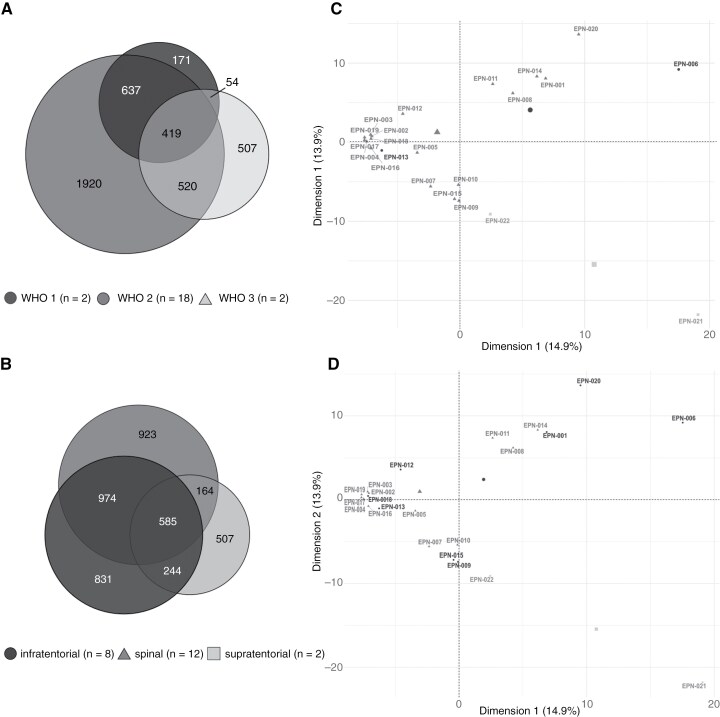
**Comparative analysis between immunopeptidomes of different EPN subgroups.** Overlap analysis of HLA class I ligand source proteins of EPNs subdivided according to (A) WHO classification and (B) tumor localization. Unsupervised PCA was performed on basis of HLA class I ligand source proteins of EPNs subdivided according to (C) WHO classification and (D) tumor localization. EPN, ependymoma; PCA, principal component analysis.

To delineate subgroup-associated differences in the immunopeptidomes of EPNs, the dataset was divided according to both WHO grading and tumor localization and used for comparative analysis. According to the current WHO classification scheme,^11^ the study cohort of the present project was categorized into 2 CNS WHO grade 1, 18 CNS WHO grade 2, and 2 CNS WHO grade 3 tumors. Besides the histopathological grading scheme, a classification system based on tumor localization and molecular genetic analyses has previously been established.^8^ The tumors of the present ependymoma cohort were further divided according to their localization into 12 spinal, 8 infratentorial, and 2 supratentorial tumors. Both supratentorial tumors were further characterized as ZFTA fusion-positive (ST-ZFTA) (former RELA fusion-positive) EPNs on the genetic level. Comparison of HLA class I immunopeptidomes from EPNs of different CNS WHO grades on source protein level revealed shared and exclusive antigens for all two subgroups (Figure 4A). A similar observation was made for comparative immunopeptidome analysis of ependymoma tumor classification according to their localization (Figure 4B). Principal component analyses (PCAs) revealed no district clustering of HLA class I ligand source proteins neither for WHO classification nor for tumor localization. (Figure 4C and D).

### Progressive EPNs show alterations of immunopeptidomes

**Figure 5.**
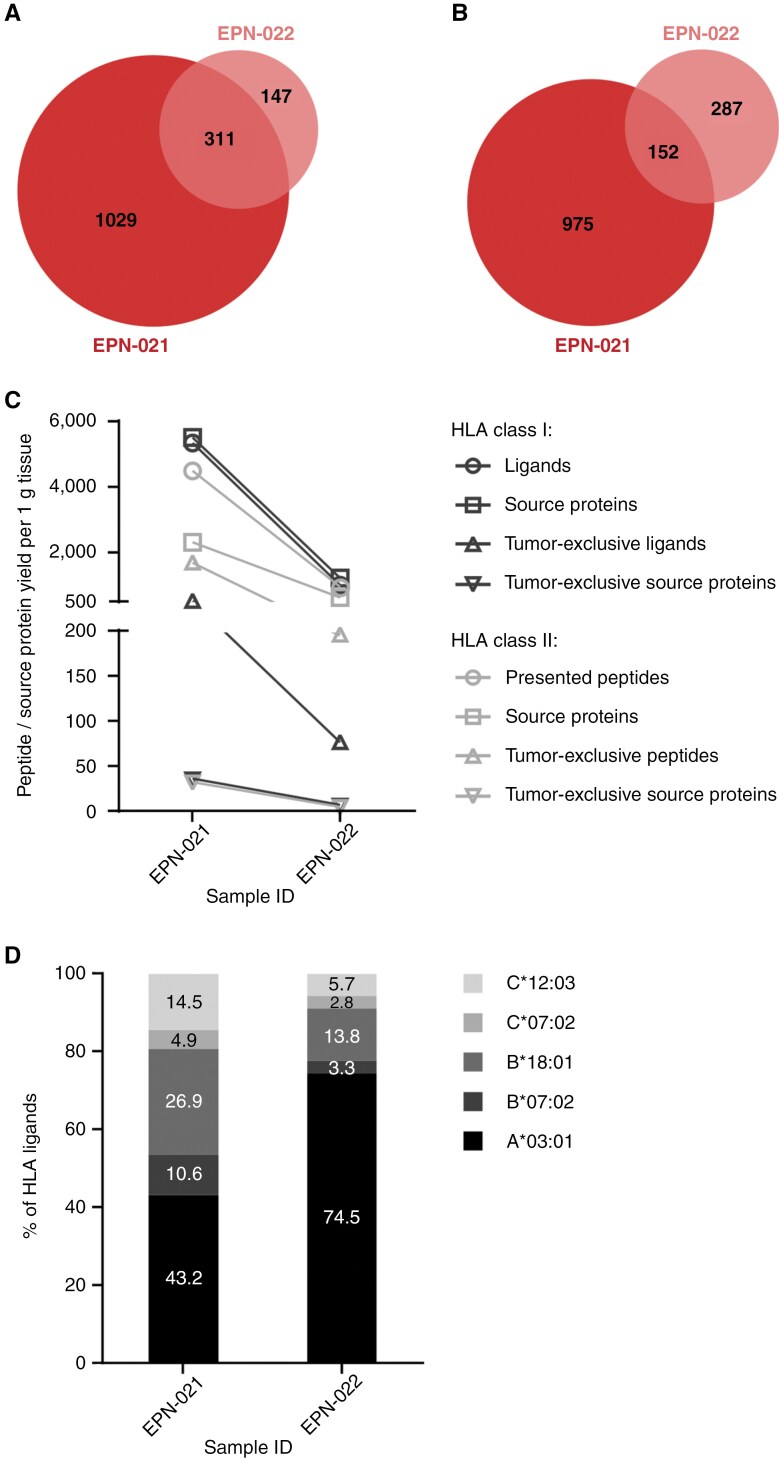
**Comparative analysis between the immunopeptidomes of two progressive ZFTA fusion-positive EPNs resected from one patient with a temporal distance of 13 months.** Overlap analysis of (A) HLA class I ligands and (B) HLA class II-presented peptides between the first recurrence (EPN-021, sample mass: 460 mg) and the second recurrence (EPN-022, sample mass: 251 mg). (C) Alterations in immunopeptidomes yields between both tumors. Yields of the second recurrence EPN-022 were normalized to the mass of the smaller sample EPN-021. (D) Distribution of HLA restrictions of HLA class I ligands in the first and the second recurrence.

The EPN cohort included two ZFTA fusion-positive (ST-ZFTA) (former RELA fusion-positive) tumors (EPN-021 and EPN-022) originating from the same patient. Whereas EPN sample EPN-021 was the first progressive tumor of this patient, the second progression (EPN-022) was surgically resected 13 months later after 6 cycles of chemotherapy treatment with carboplatin and etoposide. This sample pair enabled the investigation of potential changes on the immunopeptidomic level that emerge in relapsing tumors over time (Figure 5). Comparative analysis of the two datasets revealed shared and unique HLA-presented antigens in both tumors, and 23% of the HLA class I ligands, but only 14% of HLA class II-presented peptides detected in the first progression were also identified in the second progression (Figure 5A and B). Overall, the number of identified peptides and source proteins was lower in the second progression. This was accompanied by tumor EPN-022 presenting less tumor-exclusive peptides and source proteins than tumor EPN-021 (Supplementary Table S2; Figure 5C), which were previously defined by comparative profiling of the EPN dataset against the benign reference dataset (Figures 1D and 2D). Compared with sample EPN-021, the absolute numbers of HLA class I ligands in sample EPN-022 decreased for all HLA allotypes of the patient. However, the relative proportion of HLA-A*03:01-restricted peptides increased from 43.2% in EPN-021 to 74.5% in EPN-022, whereas a proportional loss of HLA-B*07:02, -B*18:01-, -C*07:02-, and -C*12:03-restricted peptides was observed (Figure 5D).

### Immunogenicity testing reveals spontaneous, ependymoma-specific T cell responses

**Figure 6.**
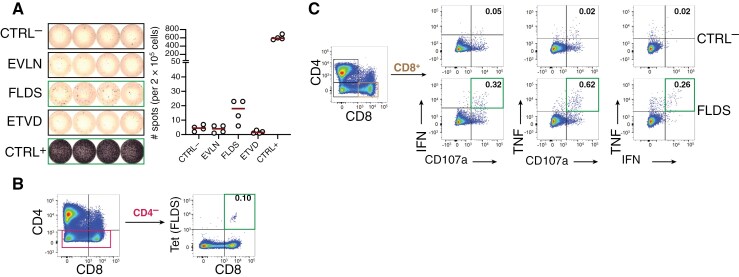
**T cell immunogenicity testing for patient EPN-018.** PBMCs were cultured for 12 days in the presence of the HLA-A*02:01 tumor ligand FLDS and IL-2 and subsequently tested for the presence of antigen-specific T cells. (A) IFN-γ ELISpot with 200.000 cells/wells seeded in 4 replicates. CTRL^-^ and CTRL^+^ were DMSO and PHA-L, respectively. Peptides EVLN and ETVD were unrelated HLA-ligands from patient EPN-014, dark red lines indicate means. (B) HLA-tetramer staining for FLDS in CD4^-^ and (C) intracellular cytokine staining after FLDS stimulation showing intracellular IFN-γ and TNF, and surface CD107a expression in CD8^+^ T cells. Frequencies of marker positive cells are given within CD8^+^ T cells and green frames indicate T cell reactivities as defined in the materials and methods section. Gating strategy is shown in Supplementary Figure S3A.

PBMCs of patient EPN-018 were obtained 6 years after surgery and tested for the presence of memory T cells against the HLA-A*02:01 restricted peptide FLDS, which had been identified on the autologous tumor. To increase the frequency of peptide-reactive T cells to a detectable level, cells were first expanded in vitro in the presence of peptide FLDS and IL-2. IFN-γ ELISpot revealed a weak but clear reactivity against FLDS (mean specific spot number: 12 per 200.000 cells) but not against two other patient-unmatched HLA-A*26 ligands EVLN and ETVD (Figure 6A). FLDS-specific T cells were also not detected in healthy donors tested in similar conditions (*n* = 8) (Supplementary Figure S2). For patient 541/16, tetramer staining on a second culture (gating strategy shown in Supplementary Figure S3A) confirmed the presence of CD8^+^ cells specific for FLDS in the blood of the patient (0.1% of the CD8^+^ subset) (Figure 6B). FLDS-specific CD8^+^ cells were polyfunctional, as assessed by upregulation of CD107a (a marker of cytotoxic degranulation) and simultaneous production of TNF and IFN-γ (Figure 6C and Supplementary Figure S3B) upon short-term peptide restimulation. The frequencies of activated CD8^+^ T cells in the ICS were higher than those detected by tetramer staining. This may indicate that some FLDS-specific T cells did not bind the tetramer construct, as already observed.^46^ In addition, reactivity of CD4^+^ cells against the same peptide was also detected in this patient (Supplementary Figure S3C).

## Discussion

In the present study, we aimed at the identification and characterization of immunogenic antigens for T-cell-based immunotherapy of EPNs. We pursued an experimental strategy including comparative immunopeptidome profiling and immunogenicity testing.

T cell recognition of HLA-presented antigens plays a central role in the immune surveillance of malignant diseases.^47,48^ Several immunotherapeutic strategies utilize respective tumor antigens to therapeutically induce an antitumor T cell response.^49–52^ In this regard, the immunopeptidome provides relevant insight into HLA-presented peptides in a defined cell population and is thus a fundamental profiling method for immunotherapeutic approaches. It enables the discovery of new peptide-specific immunotherapeutic targets. MS-based immunopeptidomics represents the only unbiased method to identify and characterize such naturally presented HLA class I- and HLA class II-restricted peptides on the cell surface.^53,54^

In recent years, immunopeptidome databases were established in various tumors such as chronic myeloid leukemia,^30^ acute myeloid leukemia,^55^ multiple myeloma,^56^ colon cancer,^57^ and ovarian cancer.^24^ Moreover, these approaches were also successfully applied to various neurooncological disease comprising meningioma, atypical teratoid rhabdoid tumors, and GBMs.^58–60^

Immunopeptidome-based discovery approaches have further led to the definition of target antigens for vaccination strategies in phase 1/2 clinical trials for hematological malignancies such as chronic lymphocytic leukemia^30^ and acute myeloid leukemia (NCT06252584) as well as in solid tumor comprising GBMs.^44,61^ The large number of recent studies analyzing immunopeptidomes to identify targets for immunotherapeutic therapies illustrates the great promise of immunotherapeutic cancer therapy, especially in incurable brain tumors.

We mapped the immunopeptidome landscape of EPNs based on the analysis of 21 EPNs. Here, a comparative HLA class I ligandome profiling of the EPN cohort against a benign reference database^30,31^ was performed to define tumor-exclusive source proteins that were identified with a representation frequency ≥10% (≥3 samples) among the EPN patients. Additionally, the comparison of our identified immunopeptidome landscape of EPNs with a previously established GBM dataset^44^ shows that EPNs and GBMs share a large set of HLA-presented antigens but also display differences. These entity-exclusive antigens are involved in cellular component and organelle organization as well as in several immunological processes. Thus, they will be particularly helpful for the selection of potential immunotherapeutic targets against EPNs. To determine their potential as immunotherapeutic targets, we additionally analyzed the immunopeptidomics of EPNs for the presence of CTA-derived HLA ligands. We identified a small panel of EPN-exclusive HLA-presented peptides (ANKRD45, ARMC3, and KDM5B) derived from previously established CTAs.^45^ CTAs are commonly expressed genes in various human tumors with a strong immunogenicity. Therefore, they are considered promising targets for immunotherapeutic approaches, such as cancer vaccines.^62^ Furthermore, we performed a comparative subgroup analysis dividing the dataset according to WHO grading and tumor localization. Of note, we did not aim here at performing complete EPN subgroup-specific analyses and immunogenicity validation because such an approach required a much larger sample size and will be realized in future studies. Our results indeed suggest potential differences between the immunopeptidomes of specific EPN subgroups. This requires further investigation with larger sample sizes. Furthermore, we had one patient in our cohort with matching samples from newly diagnosed and progressive EPNs. The single patient analysis provides first insights into immunopeptidomic alterations in progressive EPNs over time. These observations might indicate differences in terms of overall and tumor-exclusive HLA ligands, as well as the distribution of HLA class I allotype restriction between the immunopeptidomes of temporally distant tumors occurring in one patient. Considering that the patient obtained chemotherapy (carboplatin and etoposide), one explanation might be the downregulation of some HLA ligands in progressive EPNs as a form of therapy-induced resistance mechanism.^63^ As this was a single patient analysis, further studies with a larger number of cases are required to validate our findings.

Next, we performed immunogenicity testing in EPN patients and healthy donors to analyze spontaneous, ependymoma-specific T cell responses in EPN patients and tested for the presence of memory T cells against the previously identified HLA-A*02:01 restricted peptide FLDS. Although HLA-ligand specific T cells could not be identified in PBMCs obtained from two further patients (EPN-014 and EPN-12), these results demonstrate that some EPN-specific peptides identified in this study can lead to spontaneous, EPN-specific T cell responses in patients that can be detected many years postsurgery and may contribute to favorable clinical course.

In summary, the present study mapped the immunopeptidomic landscape of EPNs. To our knowledge, this study is the first report on an immunopeptidome profile of EPNs including comparative subgroup analysis and exploration of immunopeptidomic alterations in progressive EPNs. We present a set of EPN-associated HLA-presented peptides and illustrated immunopeptidome alterations in EPN-subgroups and progressive EPNs. Additionally, we demonstrated that EPN-specific antigens can lead to spontaneous, ependymoma-specific T cell responses. Our data might thus pave the way for future studies in larger networks and for clinical trials with novel immunotherapeutic strategies in this tumor entity.

## Supplementary Material

Supplementary material is available online at *Neuro-Oncology Advances* (https://academic.oup.com/noa).

vdae226_suppl_Supplementary_Material

## Data Availability

The MS raw data have been deposited to the ProteomeXchange Consortium (http://proteomecentral.proteomexchange.org) via the PRIDE partner repository^33^ with the dataset identifier PXD052448.
