# Supplementary material for: The immunopeptidomic landscape of ependymomas provides actionable antigens for T-cell-based immunotherapy
Source: Neurooncol Adv. 2025 Jan 16;7(1):vdae226. doi: 10.1093/noajnl/vdae226 (PMC12080555; doi:10.1093/noajnl/vdae226)
Supplement: vdae226_suppl_Supplementary_Material [file vdae226_suppl_supplementary_material.docx]

**Supplementary Material**

**The Immunopeptidomic Landscape of Ependymomas Provides Actionable Antigens for T Cell-Based Immunotherapy**

Lena Mühlenbruch^1,2^ †, David Rieger^2,3,4,5^ †, Hannes Becker^3,4,5,6^, Ana Maia Santos Leite^2,8^, Irina Mäurer^3,4,5^, Jens Schittenhelm^4,5,7,14^, Marissa Dubbelaar^1,2,8,9^, Leon Bichmann^8,10^, Oliver Kohlbacher^10,11,12,13^, Hans-Georg Rammensee^2,8,14^, Cécile Gouttefangeas^2,8,14^, Marcos Tatagiba^4,5,6,14^, Juliane S. Walz^1,2,14,15^* and Ghazaleh Tabatabai^2,3,4,5,14^*

^1^ Department of Peptide-based Immunotherapy, Institute of Immunology, Eberhard Karls University Tuebingen, Tuebingen, Baden-Wuerttemberg, 72076, Germany

^2^ Cluster of Excellence iFIT (EXC2180) "Image-Guided and Functionally Instructed Tumor Therapies", Eberhard Karls University Tuebingen, Tuebingen, Baden-Wuerttemberg, 72076, Germany

^3^ Department of Neurology and Interdisciplinary Neuro-Oncology, University Hospital Tuebingen, Hertie Institute for Clinical Brain Research, Eberhard Karls University Tuebingen, Tuebingen, Baden-Wuerttemberg, 72076, Germany

^4^ Center for Neuro-Oncology, Comprehensive Cancer Center Tuebingen-Stuttgart, University Hospital Tuebingen, Eberhard Karls University Tuebingen, Tuebingen, Baden-Wuerttemberg, Germany.

^5^ Center for Personalized Medicine, Eberhard Karls University Tuebingen, Tuebingen, Baden-Wuerttemberg, 72076, Germany

^6^ Department of Neurosurgery, University Hospital Tuebingen, Eberhard Karls University Tuebingen, Tuebingen, Baden-Wuerttemberg, 72076, Germany

^7^ Department of Neuropathology, University Hospital Tuebingen, Eberhard Karls University Tuebingen, Tuebingen, Baden-Wuerttemberg, 72076, Germany

^8^ Institute for Immunology, Eberhard Karls University Tuebingen, Tuebingen, Baden-Wuerttemberg, 72076, Germany

^9^ Quantitative Biology Center (QBiC), Eberhard Karls University Tuebingen, Tuebingen, Baden-Wuerttemberg, 72076 Germany

^10^ Applied Bioinformatics, Dept. of Computer Science, Eberhard Karls University Tuebingen, Tuebingen, Baden-Wuerttemberg, 72076, Germany

^11^ Cluster of Excellence Machine Learning in the Sciences (EXC2064), University of Tuebingen, Tuebingen, Baden-Wuerttemberg, 72076, Germany

^12^ Institute for Translational Bioinformatics, University Hospital Tuebingen, Tuebingen, Baden-Wuerttemberg, 72076 Germany

^13^ Institute for Bioinformatics and Medical Informatics, University of Tuebingen, Tuebingen, Baden-Wuerttemberg, 72076 Germany

^14^ German Cancer Consortium (DKTK), Partner Site Tuebingen, Tuebingen, Baden-Wuerttemberg, 72076, Germany

^15^ Clinical Collaboration Unit Translational Immunology, German Cancer Consortium (DKTK), Department of Internal Medicine, University Hospital Tuebingen, Tuebingen, Baden-Wuerttemberg, 72076, Germany

† Shared first authorship.

*Shared senior authorship

**Corresponding authors**

Juliane S. Walz, Prof. Dr. med., Department of Peptide-based Immunotherapy, University Hospital Tuebingen, Eberhard Karls University Tuebingen, phone: +49-7071-29-87305, fax:+49-7071-29-25079, e-mail: juliane.walz@med.uni-tubeingen.de

Ghazaleh Tabatabai, Prof. Dr. med. Dr. rer. nat., Department of Neurology and Interdisciplinary Neuro-Oncology, Hertie Institute for Clinical Brain Research, University Hospital Tuebingen, Eberhard Karls University Tuebingen, phone: +49-7071-29-85018, fax: +49-7071-29-5957, e-mail: ghazaleh.tabatabai@uni-tuebingen.de

**Supplementary Material: Content**

1. **Supplementary Tables 1-4**
2. **Supplementary Figures 1+2**
3. **Supplementary Data 1**

Supplementary Table 1 Overview of the ependymoma cohort and patient characteristics

22 tumor samples surgically resected from 21 different patients were included in the study. The tumors 656/15 and 974/16 originated from the same patient. f – female, m – male.

| **Sample number** | **Project ID** | **Sex** | **Age**  **at surgery** | **Diagnosis (WHO 2021)** | **CNS WHO grade** | **Location** | **Diagnosis time** | **HLA class I typing** | **HLA class II typing** |
| --- | --- | --- | --- | --- | --- | --- | --- | --- | --- |
| **1** | **EPN-001** | f | 52 | Posterior Fossa Ependymoma, NEC | 2 | infratentorial | initial | A*02:01;A*25:01  B*14:01;B*18:01  C*08:02;C*12:03 | DRB1*07:01;DRB1*15:01  DQB1*02:02;DQB1*06:02 |
| **2** | **EPN-002** | m | 17 | Spinal Ependymoma, NOS | 2 | spinal | initial | A*02:01;A*03:01  B*38:01;B*49:01  C*12:03;C*06:02 | DRB1*11:04;DRB1*16:01  DQB1*03:01;DQB1*05:02 |
| **3** | **EPN-003** | f | 42 | Spinal Ependymoma, NOS | 2 | spinal | initial | A*02:01;A*03:01  B*44:02;B*51:01  C*05:01;C*15:02 | DRB1*01:01;DRB*14:01  DQB1*05:01;DQB1*05:03 |
| **4** | **EPN-004** | m | 57 | Myxopapillary Ependymoma | 2 | spinal | initial | A*02:01;A*32:01  B*07:02;B*40:01  C*07:02;C*12:03 | DRB1*01:01;DRB1*15:01  DQB1*05:01;DQB1*06:02 |
| **5** | **EPN-005** | f | 33 | Myxopapillary Ependymoma | 2 | spinal | initial | A*02:01;A*11:01  B*15:01;B*51:01  C*03:03;C*15:02 | DRB1*04:01;DRB*12:01  DQB1*03:01 |
| **6** | **EPN-006** | m | 77 | Posterior Fossa Subependymoma | 1 | infratentorial | initial | A*03:01;A*26:01  B*35:01;B*38:01  C*04:01;C*12:03 | DRB1*13:01  DQB1*06:03 |
| **7** | **EPN-007** | m | 38 | Spinal Ependymoma, NOS | 2 | spinal | initial | A*01:01  B*08:01;B*35:02  C*07:01;C*04:01 | DRB1*04:01;DRB1*11:04  DQB1*03:02;DQB1*03:01 |
| **8** | **EPN-008** | f | 54 | Spinal Ependymoma, NOS | 2 | spinal | initial | A*11:01;A*26:01  B*44:02;B*52:01  C*05:01;C*12:02 | DRB1*13:01;DRB1*15:02  DQB1*06:03;DQB1*06:01 |
| **9** | **EPN-009** | m | 22 | Posterior Fossa Ependymoma, group B,  subclass 1 | 2 | infratentorial | initial | A*01:01;A*03:01  B*15:01;B*08:01  C*03:03;C*07:01 | DRB1*13:01;DRB1*15:02  DQB1*06:03;DQB1*06:01 |

Supplementary Table 1 continued

| **Sample number** | **Project ID** | **Sex** | **Age**  **at surgery** | **Diagnosis (WHO 2021)** | **CNS WHO grade** | **Location** | **Diagnosis time** | **HLA class I typing** | **HLA class II typing** |
| --- | --- | --- | --- | --- | --- | --- | --- | --- | --- |
| **10** | **EPN-010** | m | 30 | Spinal Ependymoma, NOS | 2 | spinal | initial | A*03:01;A*24:02  B*40:02;B*51:01  C*02:02;C*01:02 | DRB1*11:01;DRB1*13:01  DQB1*03:01;DQB1*06:03 |
| **11** | **EPN-011** | f | 45 | Spinal Ependymoma, NOS | 2 | spinal | initial | A*02:01;A*26:01  B*15:17;B*44:05  C*07:01;C*02:02 | DRB1*13:02;DRB1*16:01  DQB1*06:04;DQB1*05:01 |
| **12** | **EPN-012** | f | 67 | Posterior Fossa Ependymoma, group B, subclass 3 | 2 | infratentorial | initial | A*02:01;A*26:01  B*44:03;B*08:01  C*04:01;C*07:01 | DRB1*03:01;DRB1*07:01  DQB1*02:01;DQB1*02:02 |
| **13** | **EPN-013** | m | 71 | Posterior Fossa Subependymoma | 1 | infratentorial | initial | A*02:01;A*23:01  B*41:01;B*57:01  C*17:01;C*06:02 | DRB1*04:01;DRB1*07:01  DQB1*03:02;DQB1*03:03 |
| **14** | **EPN-014** | f | 26 | Spinal Ependymoma, NOS | 2 | spinal | initial | A*11:01;A*26:01  B*27:05;B*38:01  C*02:02;C*12:03 | DRB1*04:04;DRB1*14:01  DQB1*03:02;DQB1*05:03 |
| **15** | **EPN-015** | m | 22 | Posterior Fossa Ependymoma, group B,  subclass 4 | 2 | infratentorial | initial | A*24:03;A*30:04  B*14:02;B*18:01  C*08:02;C*07:01 | DRB1*11:04  DQB1*03:01 |
| **16** | **EPN-016** | f | 30 | Myxopapillary Ependymoma | 2 | spinal | initial | A*01:01;A*29:02  B*37:01;B*44:03  C*06:02;C*16:01 | DRB1*07:01;DRB1*10:01  DQB1*02:02;DQB1*05:01 |
| **17** | **EPN-017** | m | 52 | Myxopapillary Ependymoma | 2 | spinal | initial | A*02:01;A*30:02  B*18:01;B*39:06  C*12:03;C*05:01 | DRB1*03:01;DRB1*16:01  DQB1*02:01;DQB1*05:02 |

Supplementary Table 1 continued

| **Sample number** | **Project ID** | **Sex** | **Age**  **at surgery** | **Diagnosis (WHO 2021)** | **CNS WHO grade** | **Location** | **Diagnosis time** | **HLA class I typing** | **HLA class II typing** |
| --- | --- | --- | --- | --- | --- | --- | --- | --- | --- |
| **18** | **EPN-018** | m | 33 | Posterior Fossa Ependymoma, group B, subclass 5 | 2 | infratentorial | initial | A*02:01  B*18:01B*44:02  C*05:01 | DRB1*03:01;DRB1*15:01  DQB1*02:01;DQB1*06:02 |
| **19** | **EPN-019** | f | 53 | Spinal Ependymoma, NOS | 2 | spinal | initial | A*02:01;A*11:01  B*15:01;B*35:01  C*03:04;C*04:10 | DRB1*04:01;DRB1*07:01  DQB1*03:02;DQB1*02:02 |
| **20** | **EPN-020** | f | 52 | Spinal Ependymoma, NOS | 2 | infratentorial | recurrence | A*02:01;A*26:01  B*38:01;B*58:01 | DRB1*07:01;DRB1*13:01  DQB1*03:03;DQB1*06:03 |
| **21** | **EPN-021** | m | 41 | Supratentorial Ependymoma, ZFTA-Fusion positive | 3 | supratentorial | recurrence | A*03:01  B*07:02;B*18:01  C*07:02;C*12:03 | DRB1*04:03;DRB1*16:01  DQB1*03:05;DQB1*05:02 |
| **22** | **EPN-022** |  | 43 | Supratentorial Ependymoma, ZFTA-Fusion positive | 3 | supratentorial | recurrence |  |  |

Supplementary Table 2 Overview of HLA ligandomic yields from ependymomas

Isolated peptides were analyzed by LC-MS/MS. The HLA class I purity was calculated by the percentage of HLA class I ligands among the total number of HLA class I-presented peptides.

| **Sample number** | **Project ID** | **Tissue mass [mg]** | **HLA class I** | | | | | **HLA class II** | |
| --- | --- | --- | --- | --- | --- | --- | --- | --- | --- |
|  |  |  | **Presented peptides** | **Source proteins** | **Ligands** | **Source proteins of binders** | **Purity [%]** | **Presented peptides** | **Source proteins** |
| **1** | **EPN-001** | 248 | 735 | 793 | 636 | 700 | 87 | 249 | 169 |
| **2** | **EPN-002** | 30 | 61 | 114 | 48 | 66 | 79 | 184 | 274 |
| **3** | **EPN-003** | 184 | 189 | 263 | 179 | 202 | 95 | 153 | 149 |
| **4** | **EPN-004** | 189 | 78 | 259 | 76 | 97 | 97 | 162 | 145 |
| **5** | **EPN-005** | 137 | 505 | 684 | 441 | 508 | 87 | 160 | 177 |
| **6** | **EPN-006** | 246 | 1129 | 1113 | 1015 | 1033 | 90 | 249 | 116 |
| **7** | **EPN-007** | 158 | 872 | 1017 | 838 | 871 | 96 | 452 | 337 |
| **8** | **EPN-008** | 102 | 626 | 672 | 555 | 620 | 89 | 137 | 133 |
| **9** | **EPN-009** | 163 | 683 | 782 | 657 | 757 | 96 | 316 | 187 |
| **10** | **EPN-010** | 278 | 912 | 947 | 861 | 910 | 94 | 442 | 244 |
| **11** | **EPN-011** | 260 | 610 | 661 | 581 | 637 | 95 | 195 | 160 |
| **12** | **EPN-012** | 244 | 185 | 207 | 178 | 196 | 96 | 147 | 78 |
| **13** | **EPN-013** | 196 | 317 | 374 | 300 | 356 | 95 | 74 | 56 |
| **14** | **EPN-014** | 182 | 557 | 615 | 522 | 584 | 94 | 148 | 82 |
| **15** | **EPN-015** | 96 | 652 | 803 | 616 | 767 | 95 | 140 | 94 |
| **16** | **EPN-016** | 255 | 165 | 192 | 151 | 178 | 92 | 196 | 102 |
| **17** | **EPN-017** | 158 | 84 | 92 | 76 | 84 | 91 | 171 | 115 |
| **18** | **EPN-018** | 277 | 247 | 276 | 225 | 255 | 91 | 387 | 242 |
| **19** | **EPN-019** | 184 | 29 | 39 | 27 | 37 | 93 | 83 | 106 |
| **20** | **EPN-020** | 232 | 956 | 937 | 884 | 865 | 93 | 169 | 93 |
| **21** | **EPN-021** | 251 | 1424 | 1451 | 1340 | 1384 | 94 | 1127 | 579 |
| **22** | **EPN-022** | 460 | 481 | 699 | 458 | 562 | 95 | 439 | 288 |

Supplementary Table 3 Ependymoma-associated HLA class I ligands

After comparative profiling against the benign reference database and detailed evaluation of the resulting tumor-exclusive peptides and corresponding source proteins, six potentially tumor-associated HLA class I ligands were selected for immunogenicity testing. AC – accession; GN – gene name.

| **HLA class** | **Peptide** | | | | **Source protein** | | |
| --- | --- | --- | --- | --- | --- | --- | --- |
|  | **Sequence** | **HLA restriction** | **Representation frequency in** | | **UniProtKB AC** | **GN** | **Protein name** |
|  |  |  | **cohort** | **allotype-positive samples** |  |  |  |
| **I** | **EVIEKTSYL** | A*25:01  A*26:01 | 27% | 86% | Q9P1Z9 | CCDC180 | Coiled-coil domain-containing protein 180 |
|  | **EVLNGQVSKY** | A*25:01  A*26:01 | 27% | 86% | Q9C0G6 | DNAH6 | Dynein heavy chain 6, axonemal |
|  | **EVTERLGEF** | A*25:01  A*26:01 | 27% | 86% | Q9C0G6 | DNAH6 | Dynein heavy chain 6, axonemal |
|  | **FLDSQITTV** | A*02:01 | 27% | 50% | Q6V702 | CFAP299 | Cilia- and flagella-associated protein 299 |
|  | **ETVDENGRLY** | A*25:01  A*26:01 | 23% | 71% | Q8IYE1 | CCDC13 | Coiled-coil domain-containing protein 13 |
|  | **EVFDGTVIREL** | A*25:01  A*26:01 | 23% | 71% | Q8N1V2 | CFAP52 | Cilia- and flagella-associated protein 52 |

Supplementary Table 4 CTAs identified in the ependymoma immunopeptidome

The CTdatabase ^1^ was used as basis for the identification of CTAs. CTA – cancer-testis antigen; red – ependymoma-exclusive CTAs and CTA-derived HLA ligands.

| **HLA class** | **Peptide** | | | | **Source protein** | | | |
| --- | --- | --- | --- | --- | --- | --- | --- | --- |
|  | **Sequence** | **HLA restriction** | **Project ID** | **Representation frequency** | **UniProtKB AC** | **Gene name** | **Protein name** | **Representation frequency** |
| **I** | ALLDKLYAL | A*02:01 | EPN-012  EPN-018  EPN-013  EPN-011  EPN-020  EPN-005  EPN-003 | 32% | Q9NV31 | IMP3 | U3 small nucleolar ribonucleoprotein IMP3 | 36% |
|  | RSMEDFVTW | B*58:01 | EPN-020 | 5% |  |  |  |  |
|  | EDYTRYNQL | B*08:02;C*14:02 | EPN-015 | 5% |  |  |  |  |
|  | DVIRALAKY | A*25:01;A*26:01 | EPN-008  EPN-001  EPN-006  EPN-011  EPN-020  EPN-014 | 27% | Q5TZF3 | ANKRD45 | Ankyrin repeat domain-containing protein 45 | 27% |
|  | DPFAFIHKI | B*51:01 | EPN-005  EPN-003  EPN-010 | 14% | Q9UGL1 | KDM5B | Lysine-specific demethylase 5B | 23% |
|  | ILNPYNLFL | A*02:01 | EPN-018 | 5% |  |  |  |  |
|  | SVAQQLLNGK | A*03:01 | EPN-021 | 5% |  |  |  |  |
|  | DDWDNRTSY | B*18:01 | EPN-021 | 5% |  |  |  |  |
|  | KLPDFSWEL | A*02:01 | EPN-018  EPN-014 | 9% | Q5W041 | ARMC3 | Armadillo repeat-containing protein 3 | 14% |
|  | IINDGFYDY | A*01:01 | EPN-016 | 5% |  |  |  |  |

Supplementary Table 4 continued.

| **HLA class** | **Peptide** | | | | **Source protein** | | | |
| --- | --- | --- | --- | --- | --- | --- | --- | --- |
|  | **Sequence** | **HLA restriction** | **Project ID** | **Representation frequency** | **UniProtKB AC** | **Gene name** | **Protein name** | **Representation frequency** |
| **I** | **SEFKAMDSF** | B*40:02 | EPN-010 | 5% | P26232 | CTNNA2 | Catenin alpha-2 | 9% |
|  | **NEQDLANRF** | B*44:05 | EPN-011 | 5% |  |  |  |  |
|  | VEFPYQYDF | B*18:01 | EPN-021 | 5% | Q14667 | KIAA0100 | UPF0378 protein KIAA0100 | 9% |
|  | DYPRYLFEI | A*24:02 | EPN-010 | 5% |  |  |  |  |
|  | **RLFVTSGGLKK** | A*03:01 | EPN-009 | 5% | O75602 | SPAG6 | Sperm-associated antigen 6 | 9% |
|  | **YPEEIVRYY** | B*35:01 | EPN-006 | 5% |  |  |  |  |
|  | **SLIQKVETY** | A*02:01 | EPN-018 | 5% | **Q8TBZ0** | **CCDC110** | **Coiled-coil domain-containing protein 110** | 5% |
|  | DAVKFFVAV | B*51:01 | EPN-010 | 5% | O60271 | SPAG9 | C-Jun-amino-terminal kinase-interacting protein 4 | 5% |
|  | SFYEHIITV | B*52:01 | EPN-008 | 5% | Q5T6F0 | DCAF12 | DDB1- and CUL4-associated factor 12 | 5% |
| **II** | DPFAFIHKI | - | EPN-004  EPN-003 | 9% | Q9UGL1 | KDM5B | Lysine-specific demethylase 5B | 9% |
|  | **PFHIFKVKVTTERERMENIDSTIL** | - | EPN-012 | 5% | Q86X24 | HORMAD1 | HORMA domain-containing protein 1 | 5% |


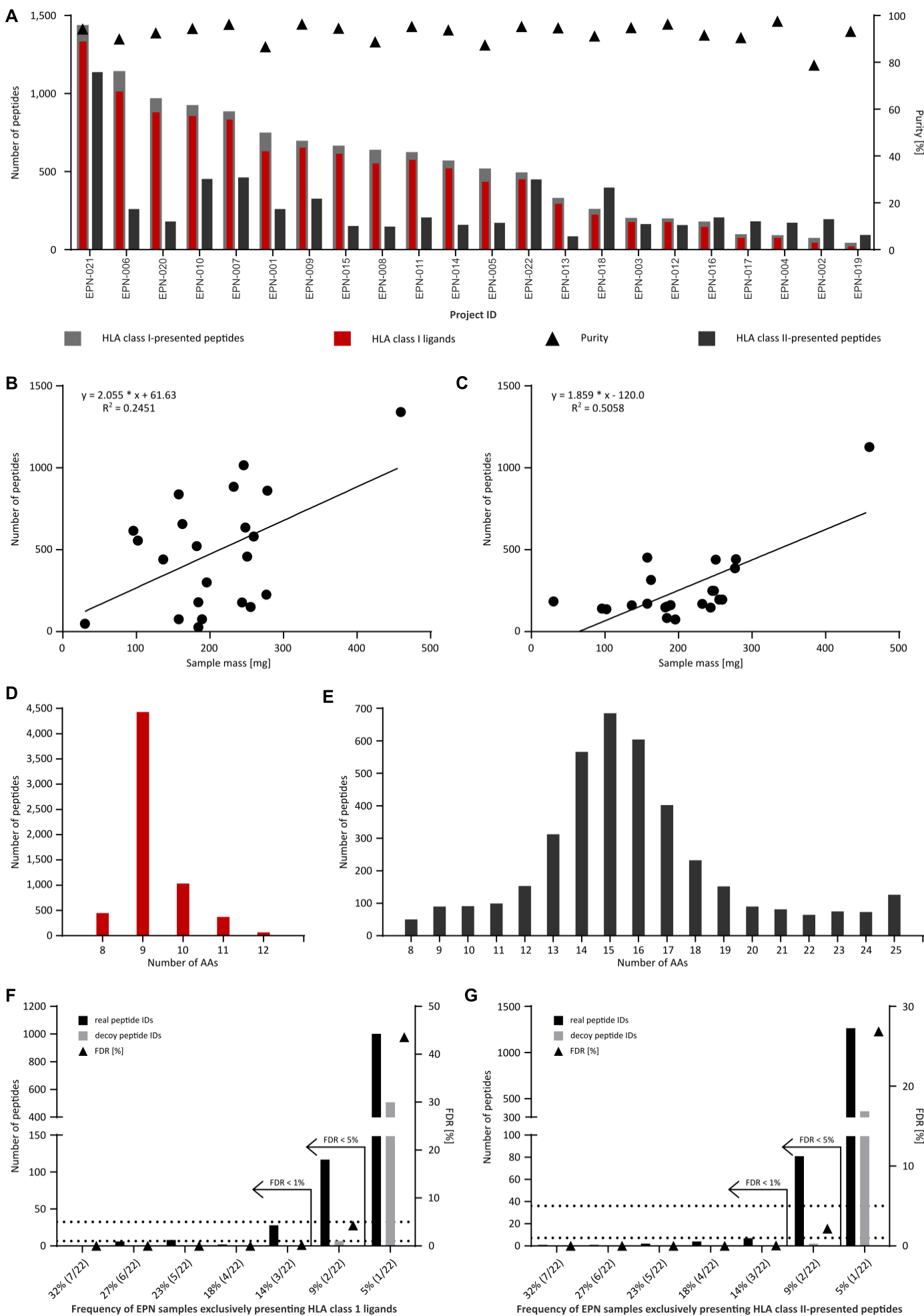


Supplementary Fig. 1

A Yields of isolated HLA class I- and class II-presented peptides for individual EPN samples achieved by LC-MS/MS analysis. Peptide yields varied between 29 and 1,424 HLA class I- (mean = 523) and between 83 and 1,127 HLA class II-presented peptides (mean = 263) per individual sample. HLA class I ligands were defined as HLA class I-presented peptides carrying a binding motif of an HLA allotype of the respective patient using SYFPEITHI (26) and NetMHCpan 4.0 (27). The purity, which is defined as the proportion of binders among all HLA class I-presented peptides, is indicated by black triangles. Correlation of sample masses and yields of B HLA class I ligands and C HLA class II-presented peptides. Length distribution analysis of D HLA class I ligands and E HLA class II-presented peptides. Based on permutation analysis, the FDRs of EPN‐associated F HLA class I and G HLA class II peptide presentation were calculated for different presentation frequencies. The process of peptide randomization, cohort assembly and tumor‐associated peptide identification was repeated 1,000 times and the mean value of resulting decoy identifications was calculated and plotted for the different threshold values together with the real peptide identifications. The corresponding FDRs for any chosen tumor‐associated peptide threshold are listed below the x‐axis. EPN – ependymoma; n(EPNs) = 22; AA – amino acid; FDR – false discovery rate.


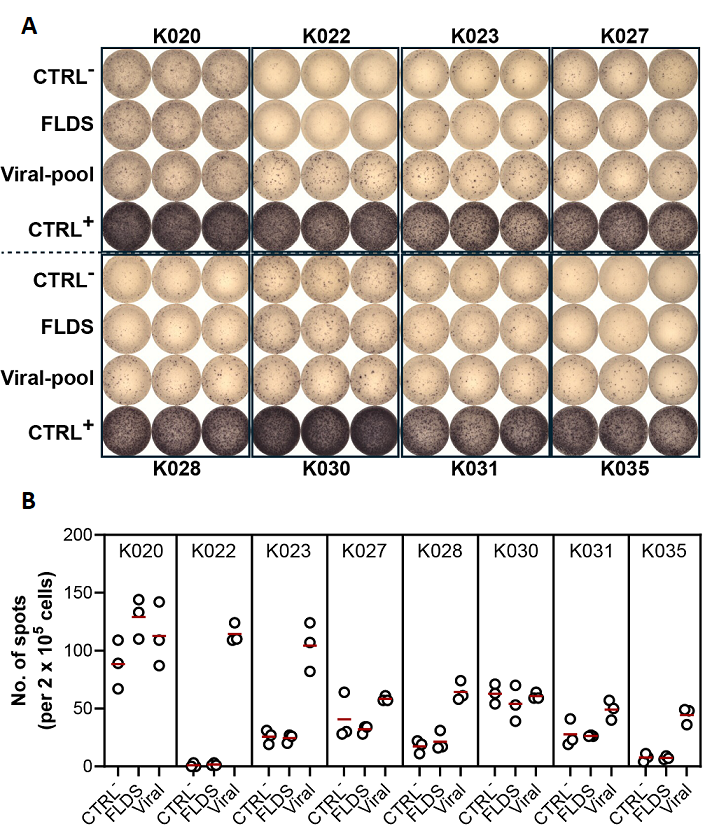


**Supplementary Fig. 2 T cell immunogenicity testing in healthy donors (HDs)**

PBMCs from 8 HDs were cultured for 12 days in the presence of the HLA-A*02:01 tumor ligand FLDS (5 µg/ml) and IL-2 and subsequently tested for the presence of antigen-specific T cells using IFN-γ ELISpot. Cells were seeded at 200 000 cells/well in 3 replicates and re-stimulated with FLDS (5 µg/ml). CTRL- and CTRL+ were DMSO and PHA-L 10 µg/ml (100.000 cells/well), respectively. Additionally, a pool of viral peptides (Influenza Matrix, CMV pp65 and EBV BMLF1, all known T cell epitopes at 1 µg/ml each) was also included as a control. **A** shows IFN-γ ELISpot plate and quantification is shown in **B**. Each dot represents one well and dark red lines indicate the mean. According to positivity criteria (mean spot numbers in test wells ≥ 2-fold the mean spot numbers in the CTRL-), no response was detected against FLDS, whereas T cell reactivity against the viral peptide pool was seen for 5 out of 8 donors (K022, K023, K028, K031 and K035).
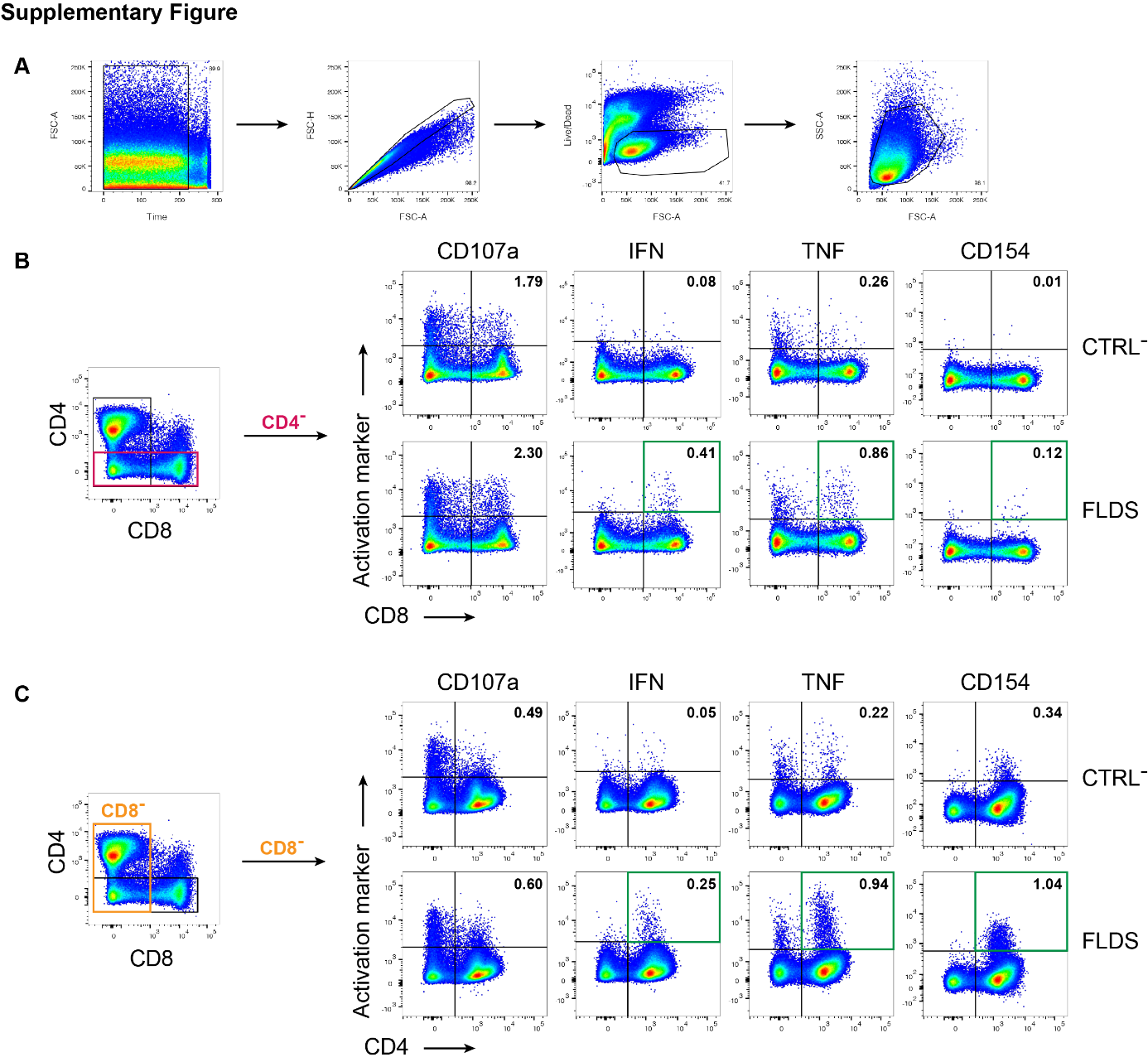


**Supplementary Fig. 3 Intracellular cytokine production in CD4^+^ and CD8^+^ cells from patient EPN-018 PBMCs after 12-day culture**

**A** Exemplary gating strategy: time gate (time / FSC-A), singlets (FSC-A / FSC-H), living cells (FSC-A / dead cell dye), lymphocytes (FSC-A / SSC-A). **B** and **C** CD107a, IFN-γ, TNF and CD154 expression in CD4^-^ cells and CD8^-^ cells, respectively. Frequencies of marker-positive cells are given within CD8^+^ and CD4^+^ T cells and green frames indicate T cell reactivities as defined in the materials and methods section.

**Supplementary Data 1**

MS spectra of ependymoma-associated HLA class I ligands and mRNA expression profiles of corresponding source proteins in healthy tissue (Genotype-Tissue Expression Project (GTEx)) ^2^. m/z – mass to charge ratio; TPM – transcripts per million.

Peptide sequence: EVIEKTSYL

Source protein: Coiled-coil domain-containing protein 180 (CCDC180)


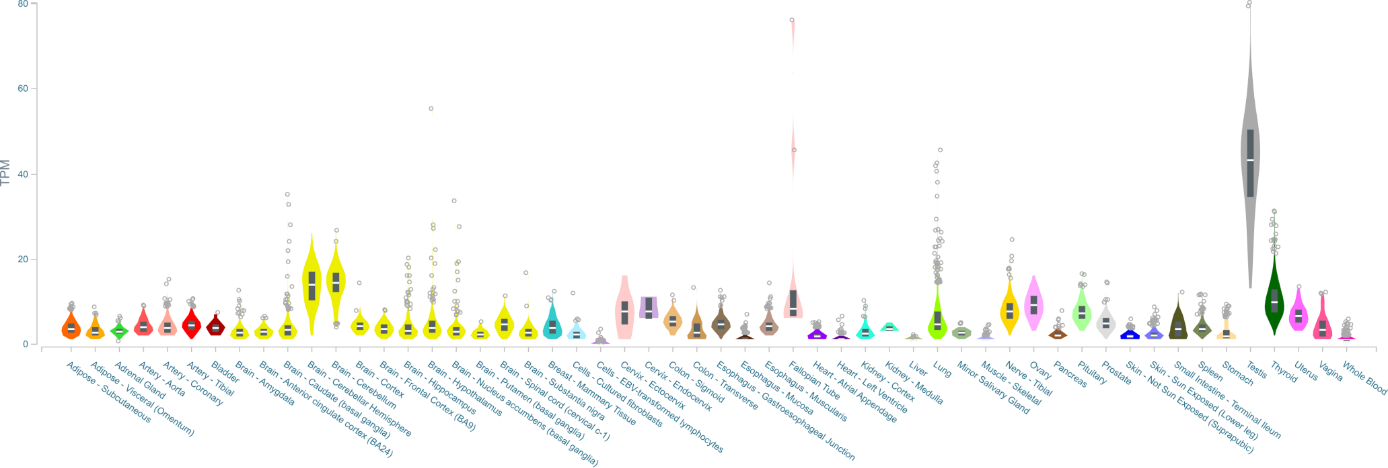


Peptide sequence: FLDSQITTV

Source protein: Cilia- and flagella-associated protein 299 (CFAP299)


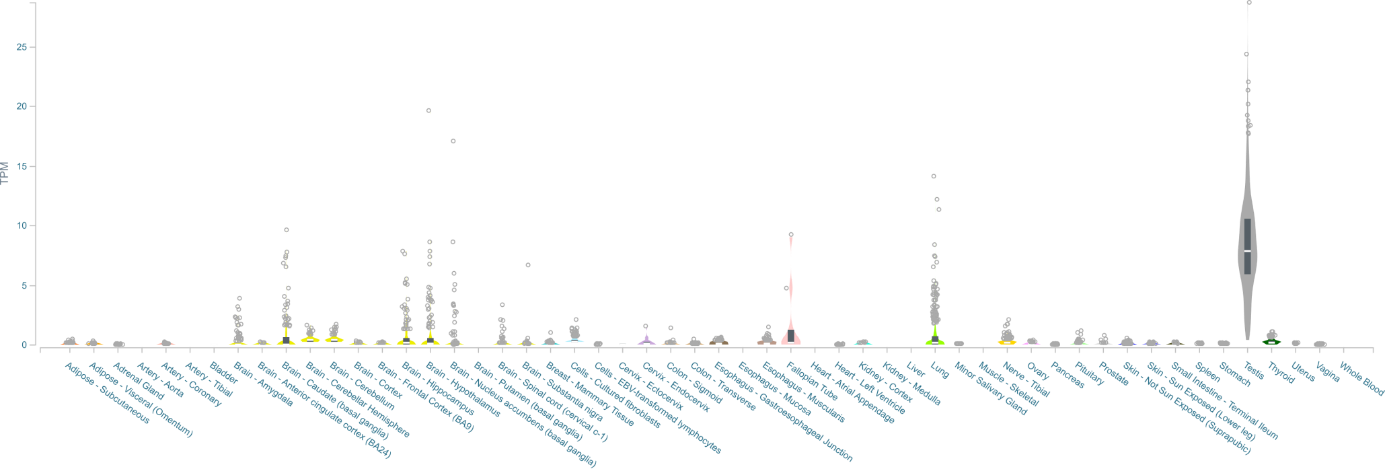


Peptide sequence: ETVDENGRLY

Source protein: Coiled-coil domain-containing protein 13 (CCDC13)


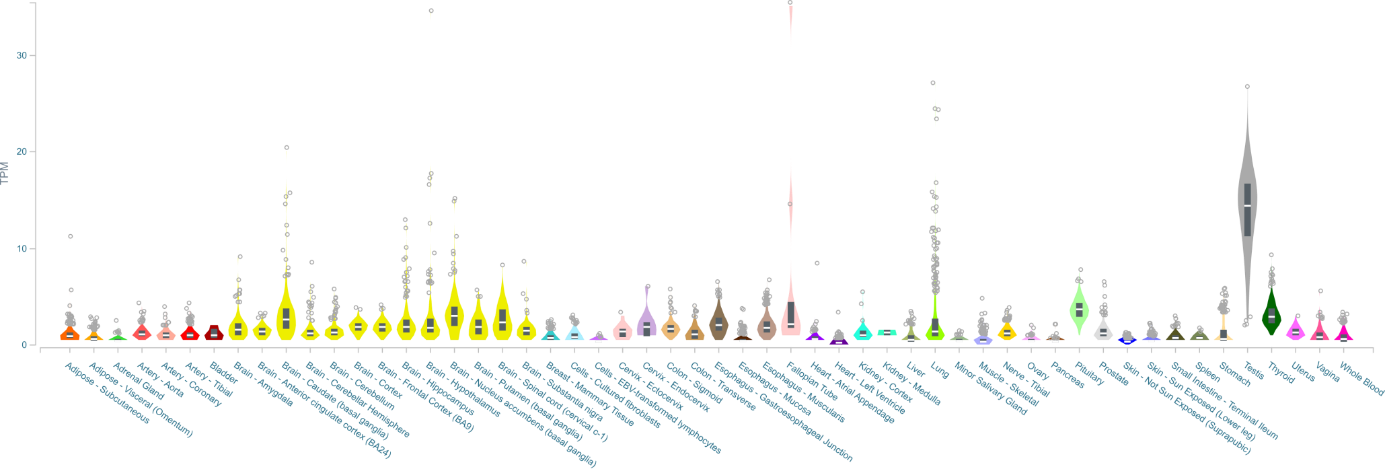


Peptide sequence: EVFDGTVIREL

Source protein: Cilia- and flagella-associated protein 52 (CFAP52)


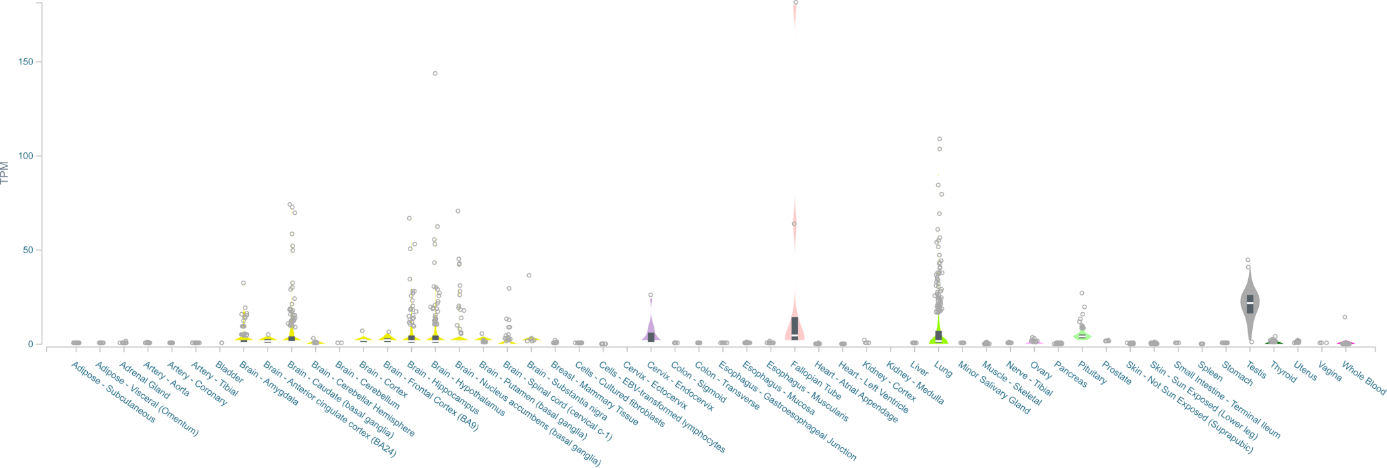


Peptide sequence: EVLNGQVSKY

Source protein: Dynein axonemal heavy chain 6 (DNAH6)


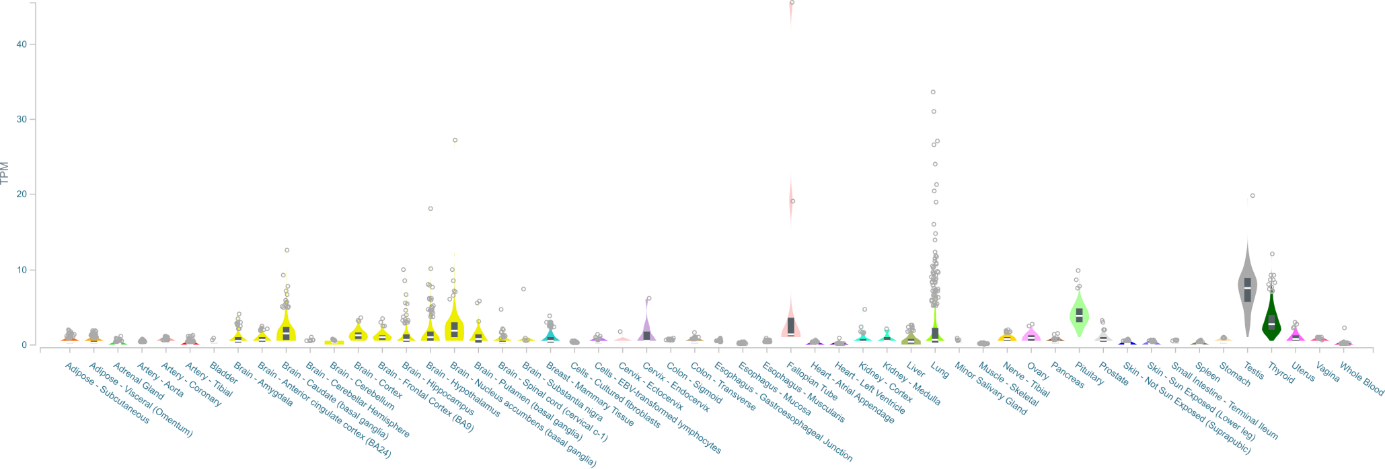


Peptide sequence: EVTERLGEF

Source protein: Dynein axonemal heavy chain 6 (DNAH6)


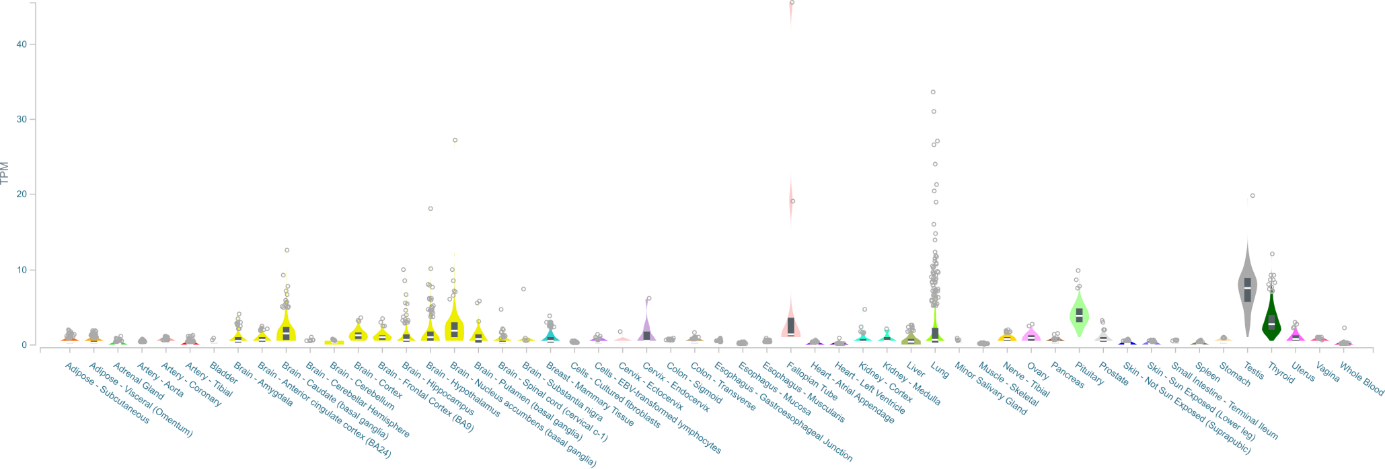


**References**

**1.** Almeida LG, Sakabe NJ, deOliveira AR, et al. CTdatabase: a knowledge-base of high-throughput and curated data on cancer-testis antigens. *Nucleic Acids Res.* 2009; 37(Database issue):D816-819.

**2.** Käll L, Canterbury JD, Weston J, Noble WS, MacCoss MJ. Semi-supervised learning for peptide identification from shotgun proteomics datasets. *Nat Methods.* 2007; 4(11):923-925.
